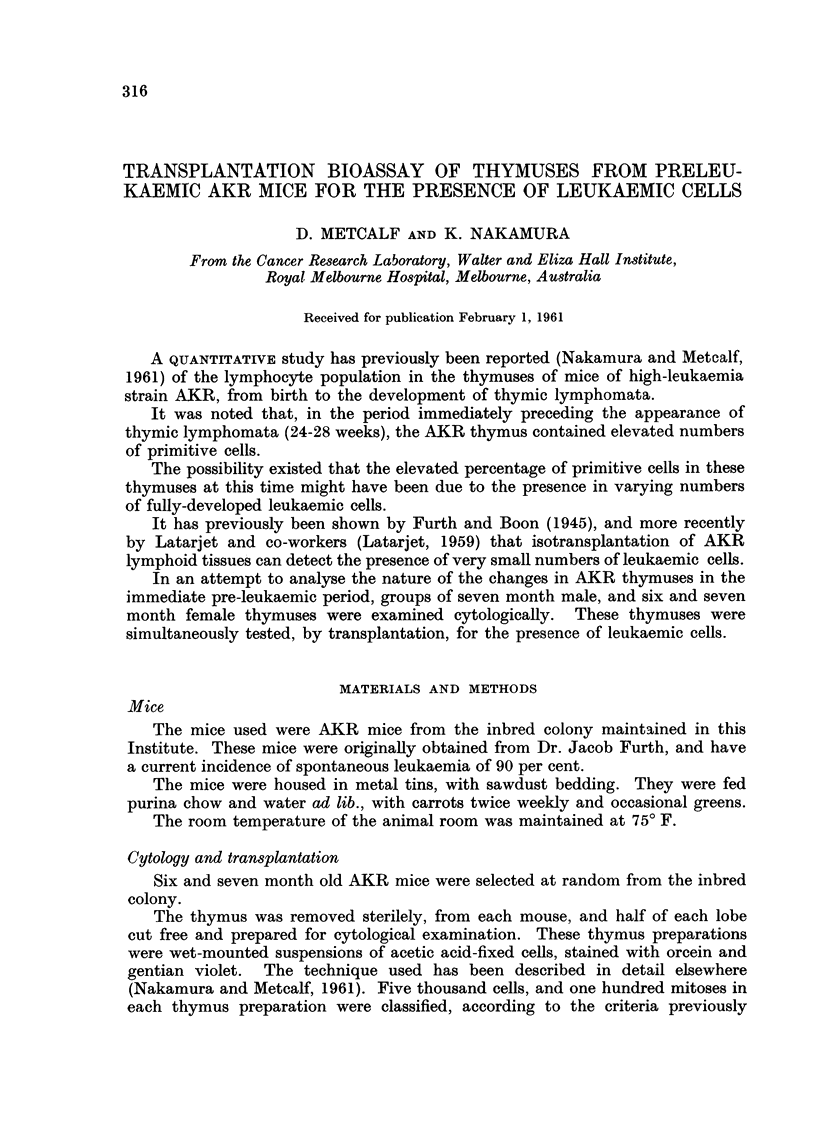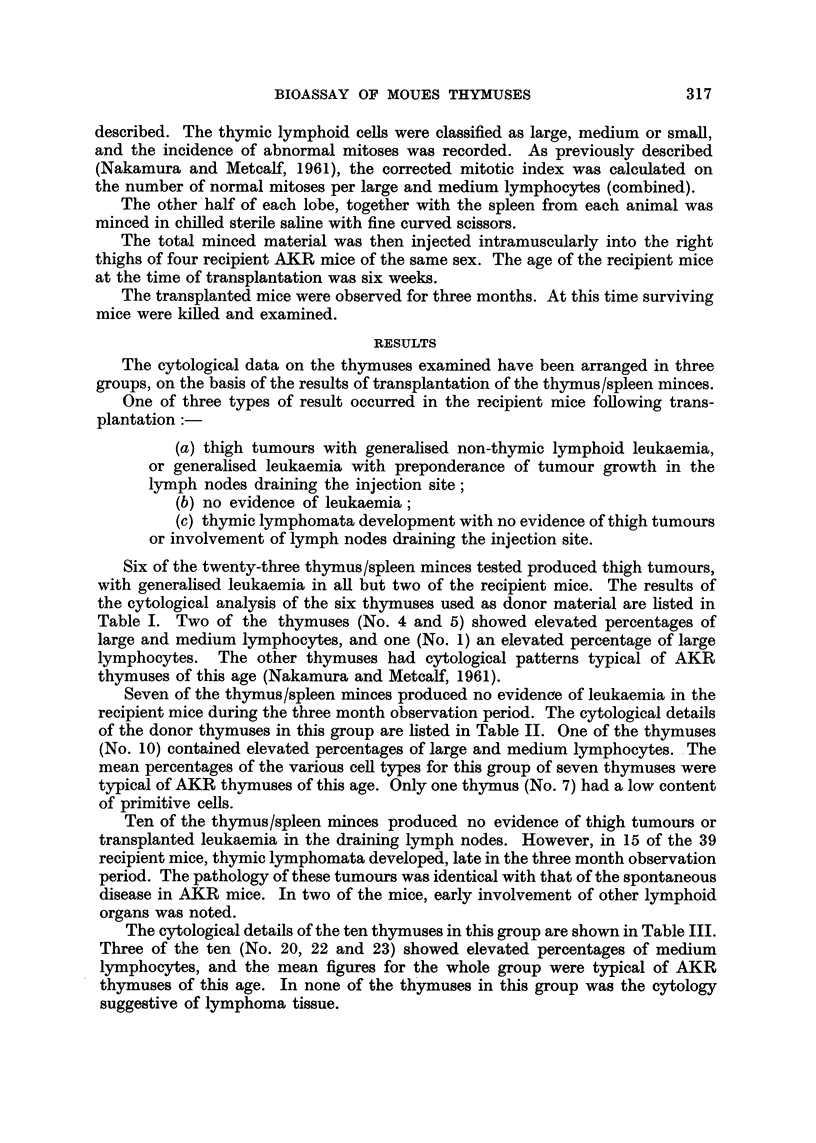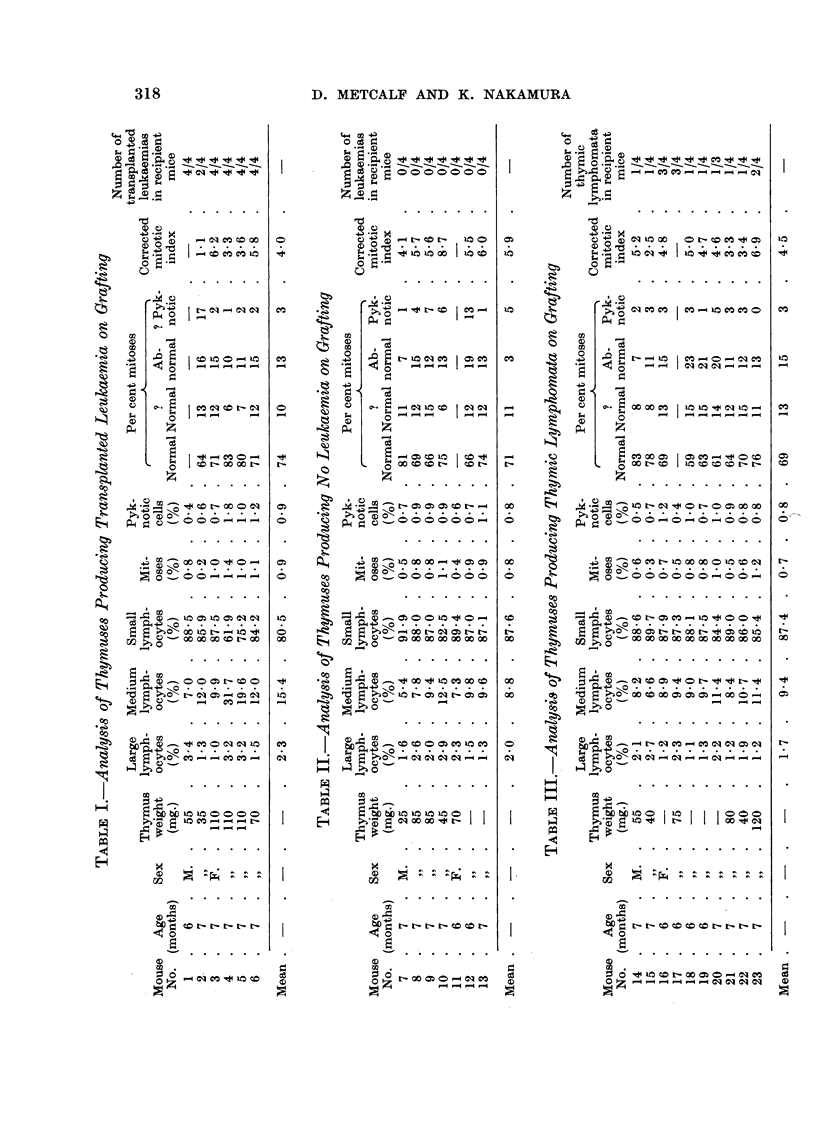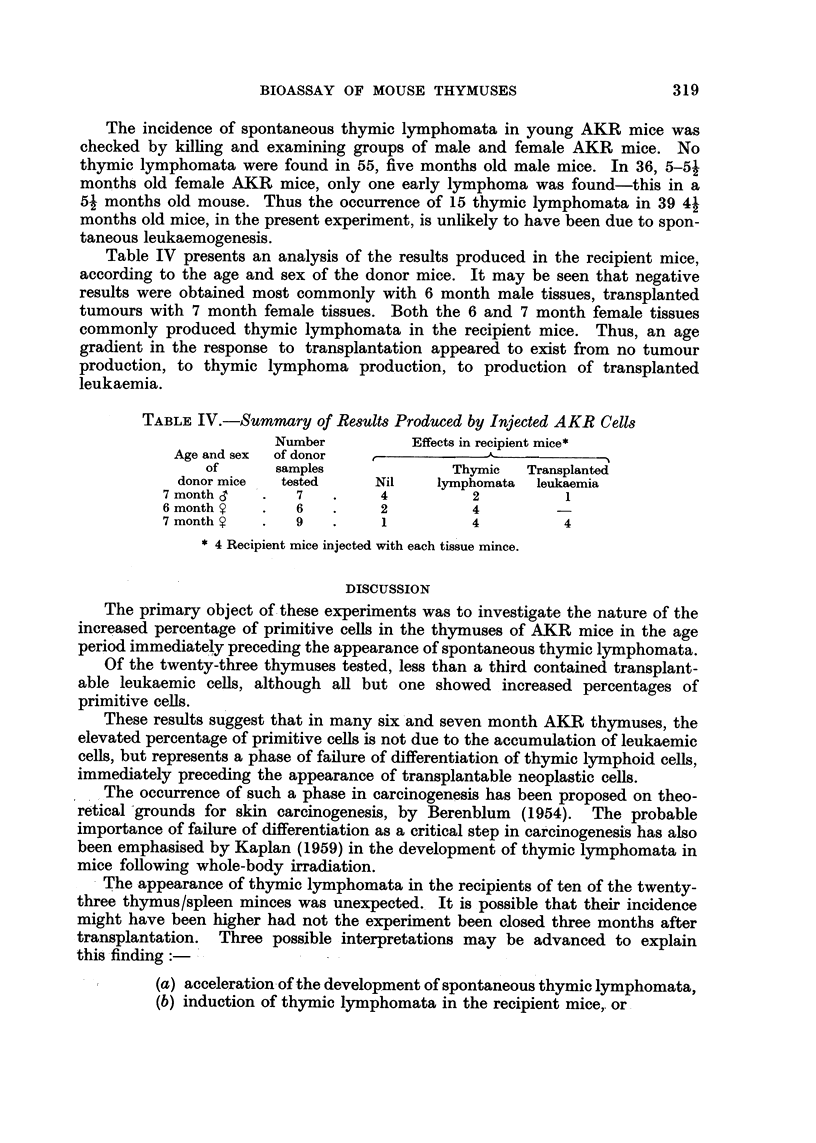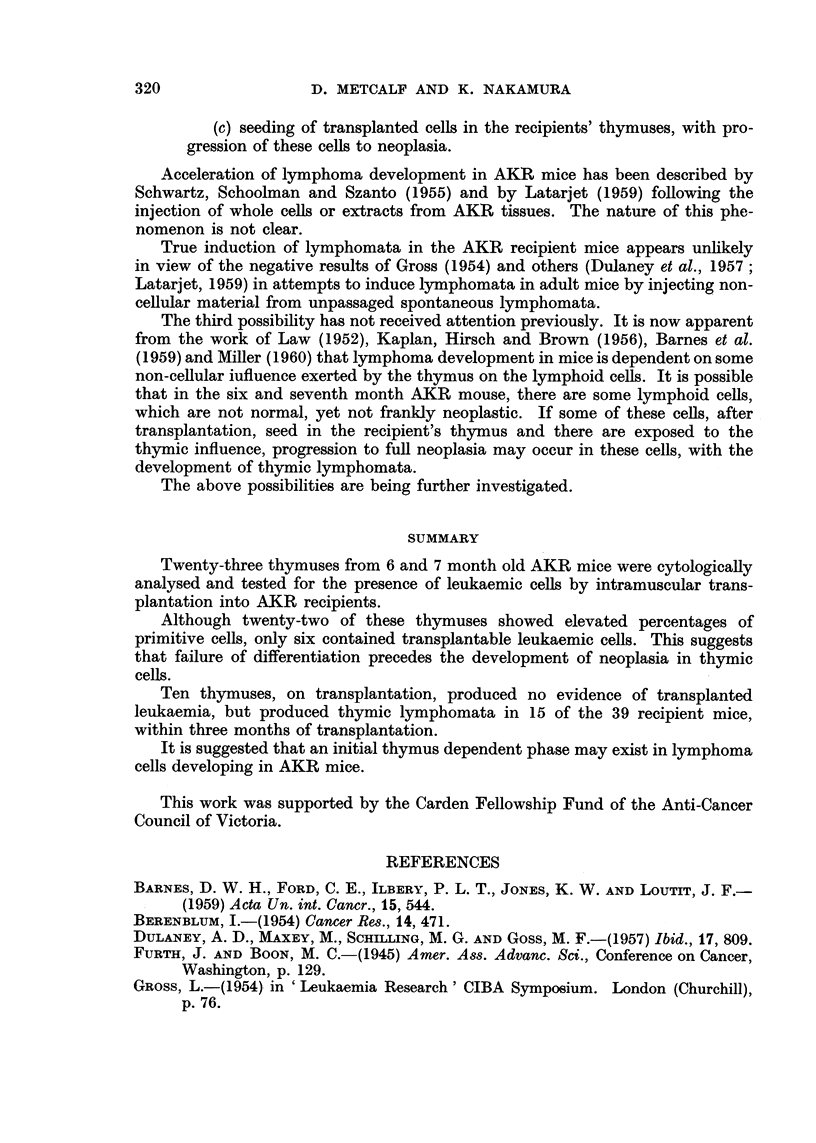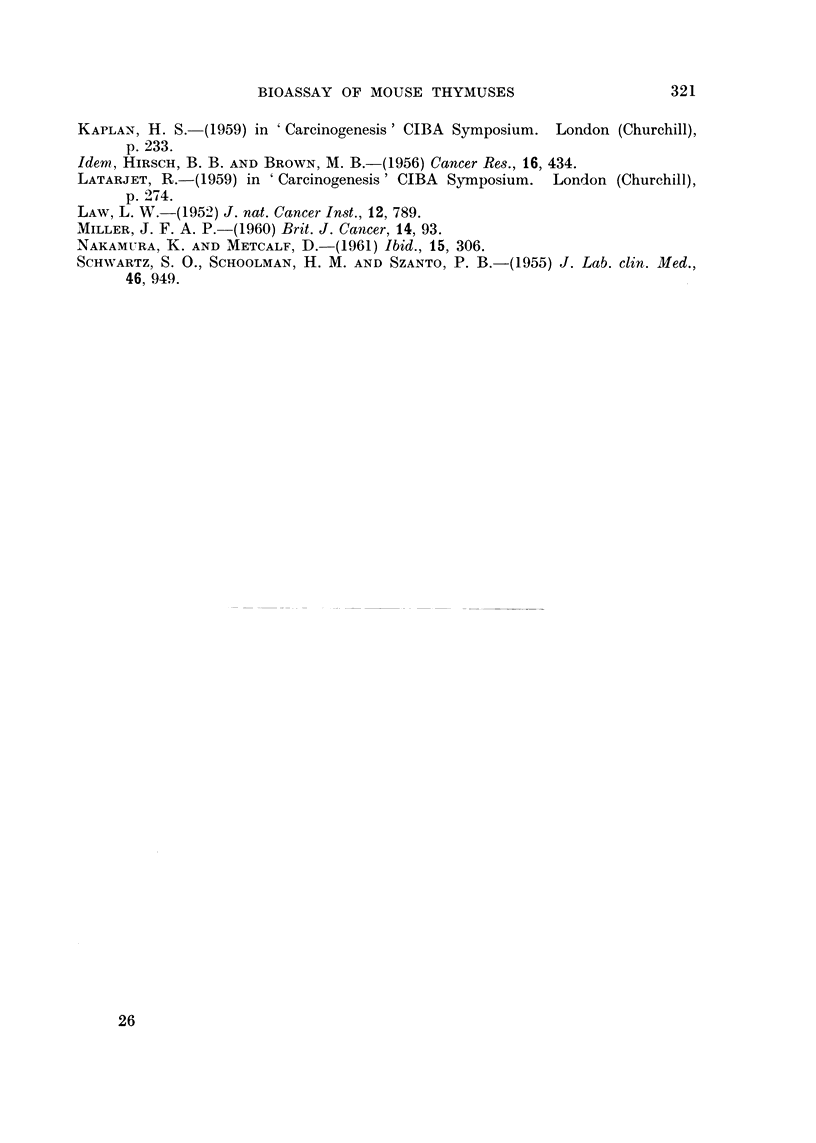# Transplantation Bioassay of Thymuses from Preleukaemic AKR Mice for the Presence of Leukaemic Cells

**DOI:** 10.1038/bjc.1961.39

**Published:** 1961-06

**Authors:** D. Metcalf, K. Nakamura


### TRANSPLANTATION BIOASSAY OF THYMUSES FROM PRELEUKAEMIC AKR MICE FOR THE PRESENCE OF LEUKAEMIC CELLS

D. METCALF AND K. NAKAMURA

*From the Cancer Research Laboratory, Walter and Eliza Hall Institute, Royal Melbourne Hospital, Melbourne, Australia*

Received for publication February 1, 1961

A QUANTITATIVE study has previously been reported (Nakamura and Metcalf, 1961) of the lymphocyte population in the thymuses of mice of high-leukaemia strain AKR, from birth to the development of thymic lymphomata.

It was noted that, in the period immediately preceding the appearance of thymic lymphomata (24-28 weeks), the AKR thymus contained elevated numbers of primitive cells.

The possibility existed that the elevated percentage of primitive cells in these thymuses at this time might have been due to the presence in varying numbers of fully-developed leukaemic cells.

It has previously been shown by Furth and Boon (1945), and more recently by Latarjet and co-workers (Latarjet, 1959) that isotransplantation of AKR lymphoid tissues can detect the presence of very small numbers of leukaemic cells.

In an attempt to analyse the nature of the changes in AKR thymuses in the immediate pre-leukaemic period, groups of seven month male, and six and seven month female thymuses were examined cytologically. These thymuses were simultaneously tested, by transplantation, for the presence of leukaemic cells.

### MATERIALS AND METHODS

### *Mice*

The mice used were AKR mice from the inbred colony maintained in this Institute. These mice were originally obtained from Dr. Jacob Furth, and have a current incidence of spontaneous leukaemia of 90 per cent.

The mice were housed in metal tins, with sawdust bedding. They were fed purina chow and water *ad lib.*, with carrots twice weekly and occasional greens.

The room temperature of the animal room was maintained at 75° F.

### *Cytology and transplantation*

Six and seven month old AKR mice were selected at random from the inbred colony.

The thymus was removed sterilely, from each mouse, and half of each lobe cut free and prepared for cytological examination. These thymus preparations were wet-mounted suspensions of acetic acid-fixed cells, stained with orcein and gentian violet. The technique used has been described in detail elsewhere (Nakamura and Metcalf, 1961). Five thousand cells, and one hundred mitoses in each thymus preparation were classified, according to the criteria previously described. The thymic lymphoid cells were classified as large, medium or small, and the incidence of abnormal mitoses was recorded. As previously described (Nakamura and Metcalf, 1961), the corrected mitotic index was calculated on the number of normal mitoses per large and medium lymphocytes (combined).

The other half of each lobe, together with the spleen from each animal was minced in chilled sterile saline with fine curved scissors.

The total minced material was then injected intramuscularly into the right thighs of four recipient AKR mice of the same sex. The age of the recipient mice at the time of transplantation was six weeks.

The transplanted mice were observed for three months. At this time surviving mice were killed and examined.

### RESULTS

The cytological data on the thymuses examined have been arranged in three groups, on the basis of the results of transplantation of the thymus/spleen minces.

One of three types of result occurred in the recipient mice following transplantation :—

(*a*) thigh tumours with generalised non-thymic lymphoid leukaemia, or generalised leukaemia with preponderance of tumour growth in the lymph nodes draining the injection site ;

(*b*) no evidence of leukaemia ;

(*c*) thymic lymphomata development with no evidence of thigh tumours or involvement of lymph nodes draining the injection site.

Six of the twenty-three thymus/spleen minces tested produced thigh tumours, with generalised leukaemia in all but two of the recipient mice. The results of the cytological analysis of the six thymuses used as donor material are listed in Table I. Two of the thymuses (No. 4 and 5) showed elevated percentages of large and medium lymphocytes, and one (No. 1) an elevated percentage of large lymphocytes. The other thymuses had cytological patterns typical of AKR thymuses of this age (Nakamura and Metcalf, 1961).

Seven of the thymus/spleen minces produced no evidence of leukaemia in the recipient mice during the three month observation period. The cytological details of the donor thymuses in this group are listed in Table II. One of the thymuses (No. 10) contained elevated percentages of large and medium lymphocytes. The mean percentages of the various cell types for this group of seven thymuses were typical of AKR thymuses of this age. Only one thymus (No. 7) had a low content of primitive cells.

Ten of the thymus/spleen minces produced no evidence of thigh tumours or transplanted leukaemia in the draining lymph nodes. However, in 15 of the 39 recipient mice, thymic lymphomata developed, late in the three month observation period. The pathology of these tumours was identical with that of the spontaneous disease in AKR mice. In two of the mice, early involvement of other lymphoid organs was noted.

The cytological details of the ten thymuses in this group are shown in Table III. Three of the ten (No. 20, 22 and 23) showed elevated percentages of medium lymphocytes, and the mean figures for the whole group were typical of AKR thymuses of this age. In none of the thymuses in this group was the cytology suggestive of lymphoma tissue.

TABLE I.—*Analysis of Thymuses Producing Transplanted Leukaemia on Grafting*

| Mouse No. | Age (months) | Sex | Thymus weight (mg.) | Large lymphocytes (%) | Medium lymphocytes (%) | Small lymphocytes (%) | Mitoses (%) | Pyknotic cells (%) | Per cent mitoses: Normal | ? Normal | Abnormal | ? Pyknotic | Corrected mitotic index | Number of transplanted leukaemias in recipient mice |
|---|---|---|---|---|---|---|---|---|---|---|---|---|---|---|
| 1 | 6 | M. | 55 | 3·4 | 7·0 | 88·5 | 0·8 | 0·4 | — | — | — | — | — | 4/4 |
| 2 | 7 | ,, | 35 | 1·3 | 12·0 | 85·9 | 0·2 | 0·6 | 64 | 13 | 16 | 17 | 1·1 | 2/4 |
| 3 | 7 | F. | 110 | 1·0 | 9·9 | 87·5 | 1·0 | 0·7 | 71 | 12 | 15 | 2 | 6·2 | 4/4 |
| 4 | 7 | ,, | 110 | 3·2 | 31·7 | 61·9 | 1·4 | 1·8 | 83 | 6 | 10 | 1 | 3·3 | 4/4 |
| 5 | 7 | ,, | 110 | 3·2 | 19·6 | 75·2 | 1·0 | 1·0 | 80 | 7 | 11 | 2 | 3·6 | 4/4 |
| 6 | 7 | ,, | 70 | 1·5 | 12·0 | 84·2 | 1·1 | 1·2 | 71 | 12 | 15 | 2 | 5·8 | 4/4 |
| Mean | — | — | — | 2·3 | 15·4 | 80·5 | 0·9 | 0·9 | 74 | 10 | 13 | 3 | 4·0 | — |

TABLE II.—*Analysis of Thymuses Producing No Leukaemia on Grafting*

| Mouse No. | Age (months) | Sex | Thymus weight (mg.) | Large lymphocytes (%) | Medium lymphocytes (%) | Small lymphocytes (%) | Mitoses (%) | Pyknotic cells (%) | Per cent mitoses: Normal | ? Normal | Abnormal | Pyknotic | Corrected mitotic index | Number of leukaemias in recipient mice |
|---|---|---|---|---|---|---|---|---|---|---|---|---|---|---|
| 7 | 7 | M. | 25 | 1·6 | 5·4 | 91·9 | 0·5 | 0·7 | 81 | 11 | 7 | 1 | 4·1 | 0/4 |
| 8 | 7 | ,, | 85 | 2·6 | 7·8 | 88·0 | 0·8 | 0·9 | 69 | 12 | 15 | 4 | 5·7 | 0/4 |
| 9 | 7 | ,, | 85 | 2·0 | 9·4 | 87·0 | 0·8 | 0·9 | 66 | 15 | 12 | 7 | 5·6 | 0/4 |
| 10 | 7 | ,, | 45 | 2·9 | 12·5 | 82·5 | 1·1 | 0·9 | 75 | 6 | 13 | 6 | 8·7 | 0/4 |
| 11 | 6 | F. | 70 | 2·3 | 7·3 | 89·4 | 0·4 | 0·6 | — | — | — | — | — | 0/4 |
| 12 | 6 | ,, | — | 1·5 | 9·8 | 87·0 | 0·9 | 0·7 | 66 | 12 | 19 | 13 | 5·5 | 0/4 |
| 13 | 7 | ,, | — | 1·3 | 9·6 | 87·1 | 0·9 | 1·1 | 74 | 12 | 13 | 1 | 6·0 | 0/4 |
| Mean | — | — | — | 2·0 | 8·8 | 87·6 | 0·8 | 0·8 | 71 | 11 | 3 | 5 | 5·9 | — |

TABLE III.—*Analysis of Thymuses Producing Thymic Lymphomata on Grafting*

| Mouse No. | Age (months) | Sex | Thymus weight (mg.) | Large lymphocytes (%) | Medium lymphocytes (%) | Small lymphocytes (%) | Mitoses (%) | Pyknotic cells (%) | Per cent mitoses: Normal | ? Normal | Abnormal | Pyknotic | Corrected mitotic index | Number of thymic lymphomata in recipient mice |
|---|---|---|---|---|---|---|---|---|---|---|---|---|---|---|
| 14 | 7 | M. | 55 | 2·1 | 8·2 | 88·6 | 0·6 | 0·5 | 83 | 8 | 7 | 2 | 5·2 | 1/4 |
| 15 | 7 | ,, | 40 | 2·7 | 6·6 | 89·7 | 0·3 | 0·7 | 78 | 8 | 11 | 3 | 2·5 | 1/4 |
| 16 | 6 | F. | — | 1·2 | 8·9 | 87·9 | 0·7 | 1·2 | 69 | 13 | 15 | 3 | 4·8 | 3/4 |
| 17 | 6 | ,, | 75 | 2·3 | 9·4 | 87·3 | 0·5 | 0·4 | — | — | — | — | — | 3/4 |
| 18 | 6 | ,, | — | 1·1 | 9·0 | 88·1 | 0·8 | 1·0 | 59 | 15 | 23 | 3 | 5·0 | 1/4 |
| 19 | 6 | ,, | — | 1·3 | 9·7 | 87·5 | 0·8 | 0·7 | 63 | 15 | 21 | 1 | 4·7 | 1/4 |
| 20 | 7 | ,, | — | 2·2 | 11·4 | 84·4 | 1·0 | 1·0 | 61 | 14 | 20 | 5 | 4·6 | 1/3 |
| 21 | 7 | ,, | 80 | 1·2 | 8·4 | 89·0 | 0·5 | 0·9 | 64 | 12 | 11 | 3 | 3·3 | 1/4 |
| 22 | 7 | ,, | 40 | 1·9 | 10·7 | 86·0 | 0·6 | 0·8 | 70 | 15 | 12 | 3 | 3·4 | 1/4 |
| 23 | 7 | ,, | 120 | 1·2 | 11·4 | 85·4 | 1·2 | 0·8 | 76 | 11 | 13 | 0 | 6·9 | 2/4 |
| Mean | — | — | — | 1·7 | 9·4 | 87·4 | 0·7 | 0·8 | 69 | 13 | 15 | 3 | 4·5 | — |

The incidence of spontaneous thymic lymphomata in young AKR mice was checked by killing and examining groups of male and female AKR mice. No thymic lymphomata were found in 55, five months old male mice. In 36, 5–5½ months old female AKR mice, only one early lymphoma was found—this in a 5½ months old mouse. Thus the occurrence of 15 thymic lymphomata in 39 4½ months old mice, in the present experiment, is unlikely to have been due to spontaneous leukaemogenesis.

Table IV presents an analysis of the results produced in the recipient mice, according to the age and sex of the donor mice. It may be seen that negative results were obtained most commonly with 6 month male tissues, transplanted tumours with 7 month female tissues. Both the 6 and 7 month female tissues commonly produced thymic lymphomata in the recipient mice. Thus, an age gradient in the response to transplantation appeared to exist from no tumour production, to thymic lymphoma production, to production of transplanted leukaemia.

TABLE IV.—*Summary of Results Produced by Injected AKR Cells*

| Age and sex of donor mice | Number of donor samples tested | Effects in recipient mice* | | |
|---|---|---|---|---|
| | | Nil | Thymic lymphomata | Transplanted leukaemia |
| 7 month ♂ | 7 | 4 | 2 | 1 |
| 6 month ♀ | 6 | 2 | 4 | — |
| 7 month ♀ | 9 | 1 | 4 | 4 |

* 4 Recipient mice injected with each tissue mince.

### DISCUSSION

The primary object of these experiments was to investigate the nature of the increased percentage of primitive cells in the thymuses of AKR mice in the age period immediately preceding the appearance of spontaneous thymic lymphomata.

Of the twenty-three thymuses tested, less than a third contained transplantable leukaemic cells, although all but one showed increased percentages of primitive cells.

These results suggest that in many six and seven month AKR thymuses, the elevated percentage of primitive cells is not due to the accumulation of leukaemic cells, but represents a phase of failure of differentiation of thymic lymphoid cells, immediately preceding the appearance of transplantable neoplastic cells.

The occurrence of such a phase in carcinogenesis has been proposed on theoretical grounds for skin carcinogenesis, by Berenblum (1954). The probable importance of failure of differentiation as a critical step in carcinogenesis has also been emphasised by Kaplan (1959) in the development of thymic lymphomata in mice following whole-body irradiation.

The appearance of thymic lymphomata in the recipients of ten of the twenty-three thymus/spleen minces was unexpected. It is possible that their incidence might have been higher had not the experiment been closed three months after transplantation. Three possible interpretations may be advanced to explain this finding :—

(*a*) acceleration of the development of spontaneous thymic lymphomata,
(*b*) induction of thymic lymphomata in the recipient mice, or

(*c*) seeding of transplanted cells in the recipients' thymuses, with progression of these cells to neoplasia.

Acceleration of lymphoma development in AKR mice has been described by Schwartz, Schoolman and Szanto (1955) and by Latarjet (1959) following the injection of whole cells or extracts from AKR tissues. The nature of this phenomenon is not clear.

True induction of lymphomata in the AKR recipient mice appears unlikely in view of the negative results of Gross (1954) and others (Dulaney *et al.*, 1957 ; Latarjet, 1959) in attempts to induce lymphomata in adult mice by injecting non-cellular material from unpassaged spontaneous lymphomata.

The third possibility has not received attention previously. It is now apparent from the work of Law (1952), Kaplan, Hirsch and Brown (1956), Barnes *et al.* (1959) and Miller (1960) that lymphoma development in mice is dependent on some non-cellular iufluence exerted by the thymus on the lymphoid cells. It is possible that in the six and seventh month AKR mouse, there are some lymphoid cells, which are not normal, yet not frankly neoplastic. If some of these cells, after transplantation, seed in the recipient's thymus and there are exposed to the thymic influence, progression to full neoplasia may occur in these cells, with the development of thymic lymphomata.

The above possibilities are being further investigated.

SUMMARY

Twenty-three thymuses from 6 and 7 month old AKR mice were cytologically analysed and tested for the presence of leukaemic cells by intramuscular transplantation into AKR recipients.

Although twenty-two of these thymuses showed elevated percentages of primitive cells, only six contained transplantable leukaemic cells. This suggests that failure of differentiation precedes the development of neoplasia in thymic cells.

Ten thymuses, on transplantation, produced no evidence of transplanted leukaemia, but produced thymic lymphomata in 15 of the 39 recipient mice, within three months of transplantation.

It is suggested that an initial thymus dependent phase may exist in lymphoma cells developing in AKR mice.

This work was supported by the Carden Fellowship Fund of the Anti-Cancer Council of Victoria.

### REFERENCES

Barnes, D. W. H., Ford, C. E., Ilbery, P. L. T., Jones, K. W. and Loutit, J. F.—(1959) *Acta Un. int. Cancr.*, **15**, 544.

Berenblum, I.—(1954) *Cancer Res.*, **14**, 471.

Dulaney, A. D., Maxey, M., Schilling, M. G. and Goss, M. F.—(1957) *Ibid.*, **17**, 809.

Furth, J. and Boon, M. C.—(1945) *Amer. Ass. Advanc. Sci.*, Conference on Cancer, Washington, p. 129.

Gross, L.—(1954) in 'Leukaemia Research' CIBA Symposium. London (Churchill), p. 76.

KAPLAN, H. S.—(1959) in 'Carcinogenesis' CIBA Symposium. London (Churchill), p. 233.
*Idem*, HIRSCH, B. B. AND BROWN, M. B.—(1956) *Cancer Res.*, **16**, 434.
LATARJET, R.—(1959) in 'Carcinogenesis' CIBA Symposium. London (Churchill), p. 274.
LAW, L. W.—(1952) *J. nat. Cancer Inst.*, **12**, 789.
MILLER, J. F. A. P.—(1960) *Brit. J. Cancer*, **14**, 93.
NAKAMURA, K. AND METCALF, D.—(1961) *Ibid.*, **15**, 306.
SCHWARTZ, S. O., SCHOOLMAN, H. M. AND SZANTO, P. B.—(1955) *J. Lab. clin. Med.*, **46**, 949.